# Role of latent tuberculosis infection on elevated risk of cardiovascular disease: a population-based cohort study of immigrants in British Columbia, Canada, 1985–2019

**DOI:** 10.1017/S0950268823000559

**Published:** 2023-04-17

**Authors:** Md. Belal Hossain, James C. Johnston, Victoria J. Cook, Mohsen Sadatsafavi, Hubert Wong, Kamila Romanowski, Mohammad Ehsanul Karim

**Affiliations:** 1 School of Population and Public Health, University of British Columbia, Vancouver, BC, Canada; 2 British Columbia Centre for Disease Control, Vancouver, BC, Canada; 3 Division of Respiratory Medicine, University of British Columbia, Vancouver, BC, Canada; 4 Faculty of Pharmaceutical Sciences, University of British Columbia, Vancouver, BC, Canada; 5 Centre for Health Evaluation and Outcome Sciences, St. Paul’s Hospital, Vancouver, BC, Canada; 6 Department of Medicine, University of British Columbia, Vancouver, BC, Canada

**Keywords:** British Columbia, cardiovascular disease, health administrative databases, latent tuberculosis infection, low tuberculosis incidence

## Abstract

We investigated cardiovascular disease (CVD) risk associated with latent tuberculosis infection (LTBI) (Aim-1) and LTBI therapy (Aim-2) in British Columbia, a low-tuberculosis-incidence setting. 49,197 participants had valid LTBI test results. Cox proportional hazards model was fitted, adjusting for potential confounders. Compared with the participants who tested LTBI negative, LTBI positive was associated with an 8% higher CVD risk in complete case data (adjusted hazard ratio (HR): 1.08, 95% CI: 0.99-1.18), a statistically significant 11% higher risk when missing confounder values were imputed using multiple imputation (HR: 1.11, 95% CI: 1.02-1.20), and 10% higher risk when additional proxy variables supplementing known unmeasured confounders were incorporated in the highdimensional disease risk score technique to reduce residual confounding (HR: 1.10, 95% CI: 1.01-1.20). Also, compared with participants who tested negative, CVD risk was 27% higher among people who were LTBI positive but incomplete LTBI therapy (HR: 1.27, 95% CI: 1.04-1.55), whereas the risk was similar in people who completed LTBI therapy (HR: 1.04, 95% CI: 0.87-1.24). Findings were consistent in different sensitivity analyses. We concluded that LTBI is associated with an increased CVD risk in low-tuberculosis-incidence settings, with a higher risk associated with incomplete LTBI therapy and attenuated risk when therapy is completed.

## Background

Over 1.9 billion people are estimated to live with latent tuberculosis infection (LTBI) globally, with 10 million people developing tuberculosis (TB) disease each year [1]. Literature has suggested that both LTBI and TB disease are associated with an increase in the risk of cardiovascular disease (CVD), which is responsible for one-third of all global deaths [2]. The increased CVD risk associated with TB disease has been reported in many cohort studies and recently summarised in two systematic reviews [3, 4]. However, the literature examining the relationship between LTBI and CVD is limited by sample size and design. In addition, no study exists in high-resource nations and low-TB-incidence settings like Canada, where immigrants and Indigenous people are disproportionately affected by TB [5].

LTBI therapy plays an important role in TB prevention as the therapy reduces the risk of developing TB disease [6]. British Columbia is a low-TB-incidence setting, with a TB incidence of 6.0 per 100,000 population [7]. Although people born outside of Canada and immigrating to British Columbia represent only 22% of the population, over 86% of people diagnosed with TB in British Columbia were born outside of Canada [7]. In British Columbia, LTBI therapy is publically funded, but treatment is voluntary [8]. As a result, many people may not initiate or complete a full course of LTBI treatment [9]. Recent studies note improvements in CVD risk factors in people taking LTBI therapy [10, 11], but the association between LTBI therapy and CVD has not been examined.

The aims of this study were twofold. First, we explored whether LTBI is associated with an increased risk of CVD among people who immigrated to British Columbia, Canada. Second, we investigated the risk of CVD associated with the completion of LTBI therapy.

## Methods

### Study setting and participants

This study is part of a larger project describing TB among foreign-born people immigrating to British Columbia [12]. For the present study, we developed a retrospective cohort of immigrants in British Columbia between 1 January 1985 and 31 December 2019. The cohort was developed based on linked immigration, public health surveillance, and health administrative databases that consist of approximately 1.4 million individuals. Data elements include immigration information, demographics, Vital Statistics (deaths in BC), Medical Services Plan (registration and physician billings), Hospital Discharge Abstract Database (inpatients and day surgeries), Statistics Canada Census (neighbourhood income quintiles), and provincial disease registries, including the Provincial TB Registry [13–21]. The setting, cohort construction, and data linkage have been described elsewhere [12].

**Figure 1.**
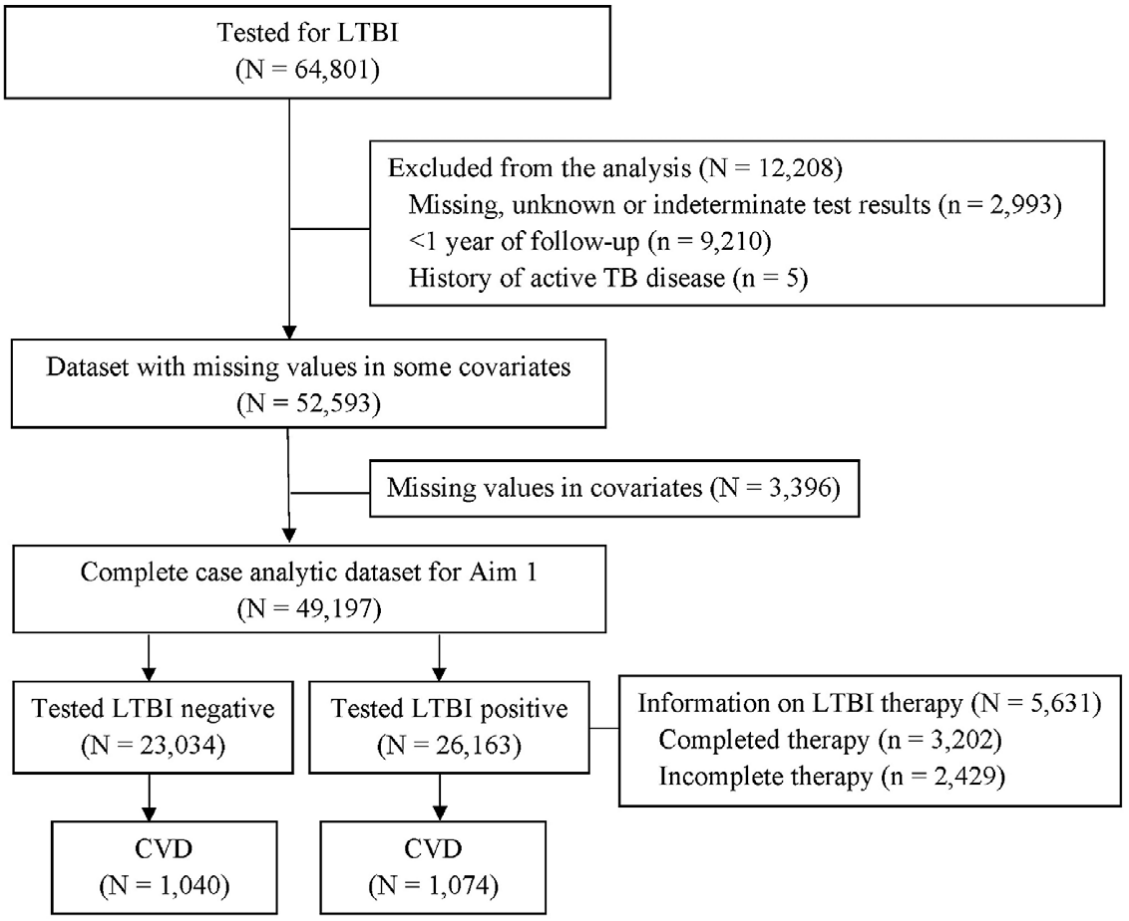
Flow chart showing the selection of the study participants who immigrated to British Columbia, Canada, between 1985 and 2019 and tested for LTBI using tuberculin skin test or interferon-gamma release assay. Here, CVD represents cardiovascular disease and LTBI represents latent tuberculosis infection.

Foreign-born persons eligible for LTBI screening include people who are in close contact with active, infectious TB, people with specific high-risk comorbidities (e.g., end-stage kidney disease, solid organ transplant, and HIV), along with people screened as part of work and school programmes. Of note, during the study period, people were not systematically screened based on county of origin or immigration classification (e.g., refugees) [7]. Screening includes assessing signs and symptoms of TB, gathering information on TB history, and evaluating the risk of developing TB. Immune testing may consist of a tuberculin skin test (TST) and/or interferon-gamma release assay (IGRA) [7]. The participants in this study were foreign-born people tested for LTBI using a TST or IGRA, as coded in the provincial TB registry. We excluded participants without valid TST or IGRA test results (Figure 1). The cohort entry date was set to 1 year after the date of residency in British Columbia was established. The date of residency was defined as 90 days before provincial health insurance coverage started or the first contact with the healthcare system [12].

### Exposures

The binary LTBI status (positive/negative) is the exposure of interest for Aim 1. Participants were classified as LTBI positive if they tested positive on TST alone or IGRA. Participants were classified as LTBI negative if they were negative on both TST and IGRA, negative on TST alone, negative on IGRA alone, and positive on TST but negative on IGRA [22]. We considered induration size ≥10 mm as TST positive and TB antigen ≥0.35 IU/ml as IGRA positive [22, 23].

The exposure variable for Aim 2 was LTBI therapy, classified as LTBI negative, LTBI positive with incomplete LTBI therapy, and LTBI positive with completed LTBI therapy. A participant would have been classified as completed LTBI therapy if they received an adequate regimen of LTBI treatment as defined by the provincial TB registry [8].

### Outcome

The outcome variable was the time from the cohort entry date to the first occurrence of a CVD event (either ischemic heart disease or stroke). The follow-up was censored at loss of medical service plan (a proxy for immigration), death, or study end (31 December 2019). The CVD events were identified from hospital separations, outpatient physician claims, and vital statistics deaths databases [24]. The details can be found on page 2 of the Supplementary Material.

### Covariates

All covariates were defined using a 1-year covariate assessment window, which began on the date residency in British Columbia was established and ended 365 days later. We identified potential confounders based on the literature and a causal diagram (see Supplementary Figure 1 on page 7). The following covariates were identified as potential confounders: age at immigration, sex, income, education, World Health Organization (WHO) region of birth, immigration class, smoking, alcohol use disorder, substance use disorder, hypertension, diabetes, chronic kidney disease, obesity, HIV/AIDS, and dyslipidemia [3, 4, 25–28]. The definition of these covariates is shown on pages 2 and 3 of the Supplementary Material.

### Statistical analysis

Descriptive statistics were calculated to assess the distribution of variables. We considered a standardised mean difference (SMD) of less than 0.2 as good covariate balancing among the exposed versus the unexposed [29]. For the Cox proportional hazards regression analyses, we reported the hazard ratio (HR) with a 95% confidence interval (CI). We used SAS 9.4 for analytic dataset preparation and R 4.2.1 for all statistical analyses.

#### Analyses for Aim 1

To determine the risk of CVD associated with LTBI, we used a Cox proportional hazards model on the complete case data. The set of covariates for adjustment was identified using the modified disjunctive cause criterion that included potential confounders and risk factors for CVD but excluded instrumental variables and mediators [30]. We adjusted the model for the following covariates: age, sex, income, education, region of birth, immigration class, alcohol use disorder, substance use disorder, hypertension, diabetes, chronic kidney disease, obesity, HIV/AIDS, and dyslipidemia.

Multiple sensitivity analyses were conducted to explore the robustness of the findings. First, we conducted a missing data analysis using multiple imputation to impute missing values in covariates. We also included a ‘tobacco use’ variable from the provincial TB registry as a proxy variable for smoking status, which is an unmeasured confounder in our analysis. The tobacco use variable was excluded from the primary analysis due to a high percentage of missing values. We imputed 10 datasets using multiple imputation with multivariate imputation via chained equation. The Cox proportional hazards model was fitted on each imputed dataset, adjusting for the covariates used in the main analysis as well as tobacco use. Second, we used the high-dimensional disease risk score [31] to reduce the bias due to unmeasured confounding by smoking [3]. The steps of high-dimensional disease risk score are described on pages 3 and 4 of the Supplementary Material. Briefly, we added the empirical/proxy variables with the covariates used in the main analysis with the assumption that empirical covariates are potentially correlated with smoking and thus can minimise bias due to unmeasured confounding by smoking [32, 33]. We used physician claims, hospital abstracts, pharmacy dispensations, and census databases to extract empirical covariates. LASSO regression was used to estimate the disease risk scores, where the predicted probabilities from the model are the disease risk scores [31]. The outcome model was the Cox proportional hazards model, adjusting for the deciles of disease risk scores.

#### Analyses for Aim 2

To investigate the risk of CVD associated with LTBI therapy, we used a Cox proportional hazards model on the complete case data, adjusting for the same set of covariates used for Aim 1.

We repeated the above-mentioned two sensitivity analyses for Aim 2. In addition, we conducted a sensitivity analysis for the potential risk of immortal time bias. We considered a time-varying LTBI therapy exposure definition and fitted the time-dependent Cox regression, adjusting for the same set of confounders used in the main analysis. The details of the sensitivity analyses can be found on pages 3–5 of the Supplementary Material.

We also conducted multiple complementary analyses for Aim 2. First, we used propensity score weighting analysis on a subset of the sample who had information on LTBI therapy to determine whether the observed association between LTBI therapy and CVD could be due to healthy user bias by comparing CVD rates before and after the LTBI test on the same subjects (i.e., subjects were self-controlled). Additionally, we conducted complementary analyses accounting for changing the exposure status for some participants due to close contact with people with TB disease and dealing with potential violations of the proportional hazards assumption (see pages 5 and 6 of the Supplementary Material).

### Ethical consideration and reporting

Ethical approval of the study was provided by the University of British Columbia (#H16-00265). We used the Reporting of Studies Conducted Using Observational Routinely Collected Health Data checklist for reporting this study (see pages 14–16 of the Supplementary Material).

## Results

### Results for Aim 1

A total of 64,801 participants had LTBI testing results (Figure 1). We excluded 12,208 participants due to invalid test results (*n* = 2,993), <1 year of follow-up (*n* = 9,210), or a history of TB disease (*n* = 5). The size of the analytic dataset with missing covariate values was 52,593. The complete case analyses included 49,197 participants, with a total of 901,734 person-years and a median time from cohort entry to CVD event or censoring of 19 years (IQR: 13–25 years). Among these participants, 26,163 (53.2%) tested LTBI positive. The mean age at cohort entry was 19.3 years, and 59.3% of participants were female. The summary statistics among those with tested LTBI negative and positive are presented in Table 1. All covariates except for education and WHO birth region were approximately balanced in terms of SMD.

**Table 1.** Characteristics of the people who immigrated to British Columbia, Canada, between 1985 and 2019 and tested for LTBI using TST or IGRA, stratified by LTBI exposure status

Characteristics	Overall (*N* = 49,197)	Tested LTBI negative (*N* = 23,034)	Tested LTBI positive^a^ (*N* = 26,163)	SMD
Total person years	901,734	423,232	478,505	
Age at immigration (years), mean (SD)	19.26 (15.76)	18.80 (16.06)	19.67 (15.48)	0.055
Females, *n* (%)	29,196 (59.3)	13,629 (59.2)	15,567 (59.5)	0.007
Education, *n* (%)				0.220
None	4,518 (9.2)	2,738 (11.9)	1,780 (6.8)	
Secondary or less	20,171 (41.0)	9,925 (43.1)	10,246 (39.2)	
Trade/diploma	8,813 (17.9)	3,732 (16.2)	5,081 (19.4)	
University degree	15,695 (31.9)	6,639 (28.8)	9,056 (34.6)	
Neighbourhood income quintile, *n* (%)				0.065
Lowest	15,937 (32.4)	7,149 (31.0)	8,788 (33.6)	
Low	12,067 (24.5)	5,648 (24.5)	6,419 (24.5)	
Middle	8,824 (17.9)	4,171 (18.1)	4,653 (17.8)	
High	6,442 (13.1)	3,122 (13.6)	3,320 (12.7)	
Highest	5,927 (12.0)	2,944 (12.8)	2,983 (11.4)	
WHO birth region, *n* (%)				0.263
Africa	1,897 (3.9)	848 (3.7)	1,049 (4.0)	
Americans	3,637 (7.4)	2,301 (10.0)	1,336 (5.1)	
Eastern Mediterranean	2,983 (6.1)	1,437 (6.2)	1,546 (5.9)	
Europe	7,750 (15.8)	3,987 (17.3)	3,763 (14.4)	
Southeast Asia	9,760 (19.8)	4,955 (21.5)	4,805 (18.4)	
Western Pacific	23,170 (47.1)	9,506 (41.3)	13,664 (52.2)	
Immigration class, *n* (%)				0.107
Economic	26,689 (54.2)	12,140 (52.7)	14,549 (55.6)	
Family	17,778 (36.1)	8,901 (38.6)	8,877 (33.9)	
Refugee	4,330 (8.8)	1,805 (7.8)	2,525 (9.7)	
Other	400 (0.8)	188 (0.8)	212 (0.8)	
Alcohol use disorder, *n* (%)	1,616 (3.3)	862 (3.7)	754 (2.9)	0.048
Substance use, *n* (%)	1,638 (3.3)	899 (3.9)	739 (2.8)	0.060
Obesity, *n* (%)	3,140 (6.4)	1,469 (6.4)	1,671 (6.4)	0.001
Hypertension, *n* (%)	1,180 (2.4)	644 (2.8)	536 (2.0)	0.049
Diabetes, *n* (%)	1,756 (3.6)	953 (4.1)	803 (3.1)	0.057
CKD, *n* (%)	132 (0.3)	95 (0.4)	37 (0.1)	0.052
HIV/AIDS, *n* (%)	660 (1.3)	386 (1.7)	274 (1.0)	0.054
Dyslipidemia, *n* (%)	326 (0.7)	144 (0.6)	182 (0.7)	0.009

Abbreviations: CKD, chronic kidney disease; HIV/AIDS, human immunodeficiency virus/acquired immunodeficiency syndrome; IGRA, interferon-gamma release assay; LTBI, latent tuberculosis infection; SD, standard deviation; SMD, standardised mean difference; TST, tuberculin skin test; WHO, World Health Organization.

a LTBI test positive was defined if the participants tested positive on TST alone or IGRA alone and defined as LTBI test negative if the participants tested negative on both TST and IGRA, negative on TST alone, negative on IGRA alone, and positive on TST but negative on subsequent IGRA.

The crude CVD rate per 100,000 person-years was 246 and 224 among people who tested LTBI positive and negative, respectively. Our fully adjusted primary analysis found an 8% higher risk of CVD among participants who tested LTBI positive compared with those who tested LTBI negative (HR: 1.08, 95% CI: 0.99–1.18) (Table 2). In our sensitivity analysis imputing missing covariate values, we observed an 11% higher risk of CVD among participants who tested LTBI positive compared with those who tested LTBI negative (HR: 1.11, 95% CI: 1.02–1.20), and the result was statistically significant. We also observed a statistically significant 10% higher risk of CVD among participants who tested LTBI positive compared with those who tested LTBI negative in the sensitivity analysis using the high-dimensional disease risk score for dealing with unmeasured confounding by smoking (HR 1.10, 95% CI: 1.01–1.20) (Table 2).

**Table 2.** Relationship between LTBI and time from the cohort entry date to the first occurrence of CVD among people who immigrated to British Columbia, Canada, between 1985 and 2019

Analysis	HR (95% CI) among people who tested LTBI positive versus negative
Unadjusted	0.91 (0.84–0.99)
Covariate adjusted (main analysis)^a^	1.08 (0.99–1.18)
Sensitivity analyses to reduce residual confounding bias associated with unavailable smoking information	
Multiple imputation after imputing and adjusting tobacco use^b^	1.11 (1.02–1.20)
High-dimensional disease risk score analysis incorporating a large number of proxies^c^	1.10 (1.01–1.20)

Abbreviations: CI, confidence interval; CVD, cardiovascular disease; HR, hazard ratio; LTBI, latent tuberculosis infection.

a The Cox proportional hazards model was fitted on the complete case dataset, adjusting for age at immigration, sex, neighbourhood income quintile, education, region of birth, immigration class, alcohol use disorder, substance use, hypertension, diabetes, chronic kidney disease, obesity, HIV/AIDS, and dyslipidemia.

b The Cox proportional hazards model was fitted on each of the 10 imputed datasets, adjusting for the same covariates used in the main analysis and tobacco use (proxy for smoking information). Rubin’s rule was used to pool the estimate. All covariates were associated with <5% missing individually (see Supplementary Figure 2), but tobacco use has more than 5% missing information, and therefore imputation was required.

c The high-dimensional disease risk score technique was used. The outcome model was the Cox proportional hazards model, adjusting for the deciles of high-dimensional disease risk scores.

### Results for Aim 2

The complete case analytic sample size for our Aim 2 was 28,665. The summary statistics were well balanced among those with LTBI therapy information (*n* = 5,631) and no LTBI therapy information (*n* = 20,532), with all SMDs <0.2 (see Supplementary Table 1 on pages 10 and 11). Among the 5,631 participants who had information on LTBI therapy, 3,202 (56.9%) completed the therapy. All covariates except for WHO birth region were approximately balanced (see Supplementary Table 2 on pages 11 and 12).

The crude CVD rate per 100,000 person-years was 230 among those who completed LTBI therapy, whereas the rate was 248 among those who did not complete LTBI therapy. Compared with those who tested LTBI negative, the fully adjusted model showed a 27% higher risk of CVD if LTBI therapy was incomplete (HR: 1.27, 95% CI: 1.04–1.55), whereas the risk was only 4% higher if LTBI therapy was complete (HR: 1.04, 95% CI: 0.87–1.24) (Table 3). The higher CVD risk in people who completed LTBI therapy remained statistically insignificant across different sensitivity analyses. However, in the sensitivity analysis dealing with missing covariate values and minimising bias due to confounding by smoking, the risk of CVD in people with incomplete LTBI therapy slightly attenuated but remained statistically significant (HR: 1.20, 95% CI: 1.06–1.35). The increased CVD risk in people with incomplete LTBI therapy also remained statistically significant in the sensitivity analyses for dealing with unmeasured confounding by smoking (HR: 1.28, 95% CI: 1.05–1.56) and dealing with potential immortal time bias (HR: 1.28, 95% CI: 1.01–1.34) (Table 3).

**Table 3.** Relationship between the completion of LTBI therapy and time from the cohort entry date to the first occurrence of CVD among people who immigrated to British Columbia, Canada, between 1985 and 2019

Analysis	HR (95% CI)	
	Complete LTBI therapy versus no LTBI	Incomplete LTBI therapy versus no LTBI
Unadjusted	0.92 (0.77–1.10)	1.01 (0.83–1.23)
Covariate adjusted (main analysis)^a^	1.04 (0.87–1.24)	1.27 (1.04–1.55)
Sensitivity analyses		
Multiple imputation after adjusting and imputing tobacco use^b^	1.05 (0.93–1.17)	1.20 (1.06–1.35)
High-dimensional disease risk score analysis incorporating a large number of proxies^c^	1.08 (0.90–1.30)	1.28 (1.05–1.56)
Addressing immortal time bias via a time-dependent Cox model^d^	1.00 (0.80–1.26)	1.28 (1.01–1.34)

Abbreviations: CI, confidence interval; CVD, cardiovascular disease; HR, hazard ratio; LTBI, latent tuberculosis infection.

a The Cox proportional hazards model was fitted on the complete case dataset, adjusting for age at immigration, sex, neighbourhood income quintile, education, region of birth, immigration class, alcohol use disorder, substance use, hypertension, diabetes, chronic kidney disease, obesity, HIV/AIDS, and dyslipidemia.

b The Cox proportional hazards model was fitted on each of the 10 imputed datasets, adjusting for the covariates used in the main analysis and tobacco use. Rubin’s rule was used to pool the estimate.

c The high-dimensional disease risk score technique was used. The outcome model was the Cox proportional hazards model, adjusting for the deciles of high-dimensional disease risk scores.

d The time-dependent Cox model was fitted, adjusting for age at immigration, sex, neighbourhood income quintile, education, region of birth, immigration class, alcohol use disorder, substance use, hypertension, diabetes, chronic kidney disease, obesity, HIV/AIDS, and dyslipidemia.

Our first complementary analysis suggests that the rate of CVD was 18% higher (rate ratio: 1.18, 95% CI: 0.83–1.69) after discontinuation of LTBI therapy among people who did not complete LTBI therapy, versus no increment in CVD rate for those who completed the LTBI therapy compared with that before the LTBI test (see Supplementary Figure 3 on page 9). In addition, results of changing the exposure status due to close contact with people with TB disease and dealing with potential violations of the proportional hazards assumption showed a similar estimate as the main analysis (see Supplementary Table 3 on page 13).

## Discussion

### Interpretations

In this study, we observed an 8% higher risk of CVD among people who tested LTBI positive than those who tested negative. Although the estimate from the primary analysis was statistically insignificant, we observed an 8%–10% higher and statistically significant association in sensitivity analyses dealing with missing values in covariates and unmeasured confounding by smoking. These findings indicate that LTBI is associated with an increased risk of CVD development in a low-TB-incidence setting.

We also observed a 27% higher CVD risk among people who did not complete LTBI therapy when compared with those who tested negative, whereas the risk was attenuated when LTBI therapy was complete. The results of sensitivity analyses for dealing with missing values in covariates, unmeasured confounding by smoking, and the potential risk of immortal time bias were not materially different compared to those derived from the main analyses.

### Contextualise the findings in the literature

Our finding of higher CVD risk associated with LTBI is consistent with findings in high-TB-incidence settings [25–28] but expands to high-income and low-TB-incidence settings. While the exact mechanism relating LTBI to CVD has not been fully elucidated, studies indicate that LTBI increases inflammation and pro-inflammatory cytokines, which may cause direct vascular damage as well as immune activation associated with LTBI [34, 35]. These results support the biological plausibility of our findings and suggest that LTBI may be a potential non-traditional risk factor for CVD.

The completion of LTBI therapy reduces the likelihood of progression to TB disease [36]. However, reducing the risk of developing TB disease may not be enough for some to decide to accept LTBI therapy. Our findings, combined with other recent literature reporting on the potential benefits of LTBI therapy beyond preventing TB disease, including reducing the risk of hyperlipidemia and hypertension [10, 11], underscore the need for further investigation into the relationship between LTBI and CVD. If confirmed, these findings may help strengthen the arguments around programmatic strategies in ensuring LTBI treatment completion.

People who did not complete LTBI therapy could have worse CVD outcomes due to potential healthy user bias, which could be one explanation for our Aim 2 results [37]. The results of our complementary analyses showed a similar estimate as the main analysis, suggesting that the observed association between LTBI therapy and CVD might not be due to healthy user bias. Notably, as we were dealing with only a subset of the whole sample in the first (also second) complementary analysis, and results from propensity score weighting were usually associated with higher variability, the above results not being statistically significant were not surprising. Even though the approach and the data for the complementary analysis were different from our main analysis, the similar direction of the findings in this complementary analysis and our primary analysis was reassuring.

### Strengths and limitations

The present study has several strengths. First, we used a large population-based longitudinal cohort that overcomes the small sample size issue in previous studies [25–28]. Second, our study overcomes the temporal limitations of previous cross-sectional studies [27, 28]. Third, we used provincial TB registry data to ascertain the LTBI exposure status and linked health administrative databases with near-complete capture of hospital encounters, physician visits, and death data to ascertain the CVD outcome status.

Despite the strengths, the study has limitations. First, there could be misclassification bias due to changes in outcome measurement, for example, CVD management improved over time. However, the study findings would be underestimated if CVD misclassification is non-differential among LTBI positive and negative [38]. Second, 53% of participants in this cohort tested LTBI positive, while the average percentage of people with LTBI positivity in migrant populations to a low-TB-incidence region is approximately 36% [39]. The higher proportion of LTBI positive in our cohort is likely due to incomplete reporting of negative LTBI screening results in the registry file. Third, unless there was close contact with people with TB disease, we assumed people were infected with LTBI before landing in British Columbia. We adjusted our regression models for overall age (i.e., age at immigration), as the LTBI diagnosis date is the arrival date. Adjusting the models for age at the LTBI testing date did not change our effect estimate as expected since age at testing was approximately balanced (in terms of SMD) among those with positive versus negative LTBI. However, information on the confounders before coming to British Columbia was unavailable. We assessed all the confounders within a year of immigration, which could bias our findings towards the null or away from the null. Fourth, smoking is an unmeasured confounder in our analysis. In particular, data have shown that people who smoke are less likely to complete LTBI therapy [40] and also at a higher risk of developing CVD. We conducted multiple sensitivity analyses attempting to minimise bias due to unmeasured confounding by smoking. The results of these sensitivity analyses were generally in the same direction as the main analyses for both Aims 1 and 2. Lastly, despite our rigorous methodology, we cannot fully exclude healthy user bias due to its observational nature.

### Implications and future scope

The findings of the present study have potential implications with 1.9 billion global LTBI cases and 523 million CVD cases [1, 41]. Recognising LTBI as a potential non-traditional risk factor for CVD and managing it could potentially reduce the future risk of developing CVD and reduce healthcare costs. Our study findings also provide evidence of the importance and benefits of completing LTBI therapy in reducing long-term adverse events such as CVD. If confirmed, the findings have substantial clinical implications on population health because of the high global prevalence of LTBI as well as the potential impact on CVD risk. This area of study may also support a patient’s decision-making around LTBI therapy. TB elimination requires programmatic strategies to engage more people in LTBI therapy, with aiming of preventing TB disease. Reducing long-term adverse events, such as CVD, could support such elimination efforts.

Immigrants from a high-TB-incidence setting must undergo testing to rule out active TB before immigrating to Canada. This most often includes sputum assessment and chest radiology. If they are diagnosed with active TB, their immigration may be delayed until they complete TB treatment and are judged to have completed an adequate course of treatment. Screening for latent TB infection (e.g., TST/IGRA) rarely occurs prior to migration to Canada. With a very low likelihood of TB transmission in Canada [42], we could argue that immigrants are likely infected with LTBI in their home country unless they have close contact with people with TB in Canada. Given the number of people migrating to Canada per year, routine latent TB screening in all immigrants requires careful evaluation and planning. A previous study by our group noted that only 4.2% of people who migrate to British Columbia and develop TB disease would be potentially ‘preventable’ if WHO LTBI screening and treatment recommendations were perfectly implemented [43].

## Conclusion

Our study suggests that LTBI is associated with an increased risk of CVD in a low-TB-incidence setting. Moreover, our results indicate that the risk of CVD is significantly higher in people who do not complete LTBI therapy but the risk is attenuated when therapy is completed. The findings were consistent in sensitivity analyses minimising bias due to unmeasured confounding by smoking and dealing with missing covariate values and immortal time bias. With the high global prevalence of LTBI, these findings require further investigation as they may have substantial clinical and programmatic implications.

## Data Availability

No primary data were collected for this study. The data from this study are held in a secure research environment managed by Population Data BC (https://www.popdata.bc.ca/). Access to data provided by the Data Steward(s) is subject to approval, but can be requested for research projects through the Data Steward(s) or their designated service providers. All inferences, opinions, and conclusions drawn in this publication are those of the author(s), and do not reflect the opinions or policies of the Data Steward(s).

## Supplementary material

For supplementary material accompanying this paper visit http://doi.org/10.1017/S0950268823000559. click here to view supplementary material
