# Supplementary material for: Role of latent tuberculosis infection on elevated risk of cardiovascular disease: a population-based cohort study of immigrants in British Columbia, Canada, 1985–2019
Source: Epidemiol Infect. 2023 Apr 17;151:e68. doi: 10.1017/S0950268823000559 (PMC10204137; doi:10.1017/S0950268823000559)
Supplement: Supplementary file 1 [file S0950268823000559sup001.pdf]

Appendix "Role of latent tuberculosis infection on elevated risk of cardiovascular disease: a population-based cohort study of immigrants in British Columbia, Canada, 1985-2019"

0. Abbreviations

CI	Confidence interval
CKD	Chronic kidney disease
CVD	Cardiovascular disease
HIV/AIDS	Human immunodeficiency virus/acquired immunodeficiency syndrome
HR	Hazard ratio
LTBI	Latent tuberculosis infection
SMD	Standardize mean difference
WHO	World Health Organization

1. Outcome definition

The outcome variable was the time from cohort entry date to the first occurrence of CVD (composite of ischemic heart disease or stroke) or censoring (end of provincial health insurance coverage as a proxy for emigration, death due to other than CVD, or study end). The CVD events were identified from hospital separations, outpatient physician claims, and vital statistics deaths databases proposed by Tonelli et al. [1]. The case definition of ischemic heart disease includes 1 hospitalization or underlying cause of death with ICD-9 codes 410.x–414.x or ICD-10 codes I20.x–I25.x; the case definition of stroke includes 1 hospitalization or 1 visit to a health professional or underlying cause of death with ICD-9 codes 362.34, 430.x–438.x or ICD-10 codes G45.x, G46.x, H34.0, I60.x–I69.x [1,2].

2. Covariate definition

Variable	Definition
Age at immigration	Continuous
Sex	Binary (female, male)
Income	The categorical neighbourhood income quintile was defined as the lowest 20%, second lowest 20%, middle 20%, second highest 20% and highest 20%.
Education	Categorical (none or no education, secondary or less, trade or diploma, and university degree)
World Health Organization (WHO) region of birth	The categorical WHO birth region was defined as Africa, Americans, Eastern Mediterranean, Europe, Southeast Asia, and Western Pacific [3,4].
Immigration class	The categorical immigration class was defined as economic class, family class, refugee, and others.
Smoking	Unmeasured
Alcohol use disorder	The binary alcohol use disorder was defined using the alcohol abuse variable in the TB registry files(yes/no), ICD-9 code (2652, 2911, 2912, 2913, 2915, 2918, 2919, 3030, 3039, 3050, 3575, 4255, 5353, 5710, 5711, 5712, 5713, 980, V113) and ICD-10 code (F10, E52, G621, I426, K292, K700, K703, K709, T51, Z502, Z714, Z721).
Substance use	The binary substance use was defined using the ICD-9 code (292, 304, 3052, 3053, 3054, 3055, 3056, 3057, 3058, 3059, V6542) and ICD-10 code (F11, F12, F13, F14, F15, F16, F18, F19, Z715, Z722).

Hypertension	The binary hypertension was defined using the ICD-9 code (402-405) and ICD-10 code (I11-I15).
Diabetes	The binary diabetes was defined using the ICD-9 code (2405-2509) and ICD-10 code (E102-E148).
CKD	The binary CKD was defined using CKD variable in the Renal Agency database (≥1 chronic dialysis records or any glomerular filtration rate (GFR) <30 ml/min) and the ICD-9 code (584-586) and ICD-10 code (N17-N19).
Obesity	The binary obesity was defined using the ICD-9 code (2780) and ICD-10 code (E66).
HIV/AIDS	The binary HIV/AIDS was defined using the BC HIV/AIDS datafiles (positive/negative), ICD-9 code (042-044) and ICD-10 code (B20-B23, B24).
Dyslipidemia	The binary dyslipidemia was defined using the ICD-9 code (272) and ICD-10 code (E78).

3. Description of sensitivity analyses for Aim 1

Dealing with missing values in covariates

In our primary analysis using complete case dataset, we excluded 3,396 participants (~6.5%) due to missing data in covariates. Particularly, we have 3.7% of missing values for WHO region of birth, followed by income (2.1%), education (0.6%), and immigration class (0.1%) (Appendix Figure 2; pp 8). We used multiple imputation to impute those missing values by considering the missing at random assumption. We also added the 'tobacco use' variable from the TB registry and imputed the missing values for that variable. Since we were dealing with time-to-event outcomes, predictors used to build the imputation model included all covariates used in the main analysis, tobacco use, LTBI exposure status, CVD outcome event, and the Nelson–Aalen estimator of CVD event [5]. We imputed 10 datasets with five iterations. The Cox proportional hazards model was fitted on each imputed dataset, adjusting for the covariates used in the main analysis. Finally, we pooled the estimates using Rubin's rule [6].

Dealing with unmeasured confounding by 'smoking'

We used the high dimensional disease risk score to minimize bias due to unmeasured confounding [7]. There were seven steps of high dimensional disease risk score:

- Step 1 – identify the source of empirical/proxy variables: All empirical covariates were identified in a one-year covariate assessment window prior to the cohort entry date. The following data sources were used:
    - Physician claims database for ICD-9 diagnostic codes

  - Hospital abstracts database for ICD-9 and ICD-10 diagnosis codes, procedure codes, and intervention codes
  - Pharmacy dispensations database for the drug identification number, generic names, American hospital formulary codes, Pharmacare therapeutic class
  - Census database for income band.
- Step 2 – empirical variable identification: Based on the prevalence, the 200 most prevalent codes in each data dimension were considered.
- Step 3 – assessing recurrence of codes: We generated three binary recurrence covariates for each of the candidate empirical covariates: (i) once, (ii) frequent, and (iii) sporadic.
- Step 4 – prioritizing covariates: We used the Bross formula to prioritize the covariates.
- Step 5 – variable selection: We selected the top 200 variables based on the log of bias calculated in step 4.
- Step 6 – predicting disease risk scores: In this step, we fitted the outcome model with investigator-specified variables (all confounders used in the main analysis) and empirical variables from step 5 on the cohort with only LTBI negative. Then we fitted the LASSO model (binary CVD as the outcome) to deal with overfitting of the model [7]. Hyperparameters of the model were chosen using 5-fold cross-validation. The disease risk scores are the predicted probabilities from the LASSO model.
- Step 7 – outcome modelling: The outcome model was the Cox proportional hazards model, adjusting for the deciles of disease risk scores. We used a robust sandwich-type variance estimator to estimate the 95% CI.

4. Description of sensitivity analyses for Aim 2

Dealing with missing values in covariates

The same as Aim 1.

Dealing with unmeasured confounding by 'smoking'

We used the high dimensional disease risk score to minimize bias due to unmeasured confounding in Aim 2 [7]. There were seven steps of high dimensional disease risk score, with steps 1-3 are identical to the steps defined for Aim 1. Steps 4 to 7 are as follows:

- Step 4 – prioritizing covariates: We used the hybrid LASSO method to prioritize the covariates [8]. The 5-fold cross-validation was used to choose the hyperparameters of the model.
- Step 5 – variable selection: We selected the top 200 variables based on the log of bias calculated in step 4.
- Step 6 – predicting disease risk scores: We fitted the outcome model (CVD as the outcome) on the cohort with only LTBI unexposed, with the investigator-specified variables (all confounders used in the main analysis) and empirical variables from step 5.

We estimated the disease risk scores by fitting LASSO regression. We used 5-fold cross-validation to choose the hyperparameters of the model.

- Step 7 – outcome modelling: The outcome model was the Cox proportional hazards model, with categorical LTBI therapy as the exposure and deciles of disease risk scores as a covariate. Again, we used a robust sandwich-type variance estimator to estimate the 95% CI.

Dealing with potential immortal time bias

As a sensitivity analysis for the potential risk of immortal time due to defining LTBI therapy exposure at the cohort entry date, we conducted a sensitivity analysis with a time-varying LTBI therapy exposure definition. The unexposed time for those with LTBI therapy information was the time from cohort entry date to the starting date of LTBI therapy. The exposed time began at the LTBI therapy starting date and continued until an event or censoring date was reached. On the other hand, the exposure time for those without LTBI was the time from the cohort entry date to the date of an event or censoring. We fitted the time-dependent Cox regression [9], adjusting for the same set of confounders used in the main analysis.

5. Description of complementary analyses for Aim 2

First, in the search for reducing healthy user bias in the association between LTBI therapy and CVD, we used propensity score weighting analysis on a subset of the sample who had information on LTBI therapy. Although subjects were self-controlled, the subjects who developed CVD before the test were omitted from the post-test calculation. Thus, adjusted rates were deemed more appropriate than crude estimates. The propensity score weighting approach was used to adjust for measured confounding among those who completed the LTBI therapy versus did not complete the therapy (adjusting for the same confounders used in the main analysis). Logistic regression was used to estimate the propensity scores. The mean stabilized weight was 1, with a minimum of 0.79 and a maximum of 1.54. The CVD rate ratio was calculated by re-weighting each participant's contribution by the stabilized inverse probability weights.

Second, we compared the medication adherence rate for two comorbidities (e.g., metformin, insulin, sulfonylurea for diabetes, aspirin for anti-inflammation). We considered SMD less than 0.2 as a good balance of adherence rate among those who completed versus did not complete the LTBI therapy.

Third, for the main analysis, we assumed LTBI status to be positive or negative from the cohort entry date. However, 53 participants had LTBI status changed (from negative to positive) due to close contact to people with TB disease. To account for changing the exposure status due to close contact, we conducted our third complementary analysis and used a time-varying LTBI exposure definition. The unexposed time for those who had close contact was the time from cohort entry date to the date of contact. Since the exact date of contact was unknown, we considered the unexposed time as the time from the index date to the date of the LTBI test. The exposed time

began at the date of the LTBI test and continued until an event or censoring date was reached. On the other hand, the exposure time for those without close contact was the time from the cohort entry date date to the date of an event or censoring. The time-dependent Cox model was fitted, adjusting for the same confounders used in the main analysis.

Fourth, the proportional hazards assumption was violated for age, birth region, immigration class, substance use, and chronic kidney disease. To deal with that problem, the modified Poisson regression with binary CVD outcome variable and an offset by the natural logarithm of follow-up time was fitted, adjusting for the same confounders used in the main analysis. The 95% confidence interval was calculated using the robust sandwich method.

6. Appendix figures

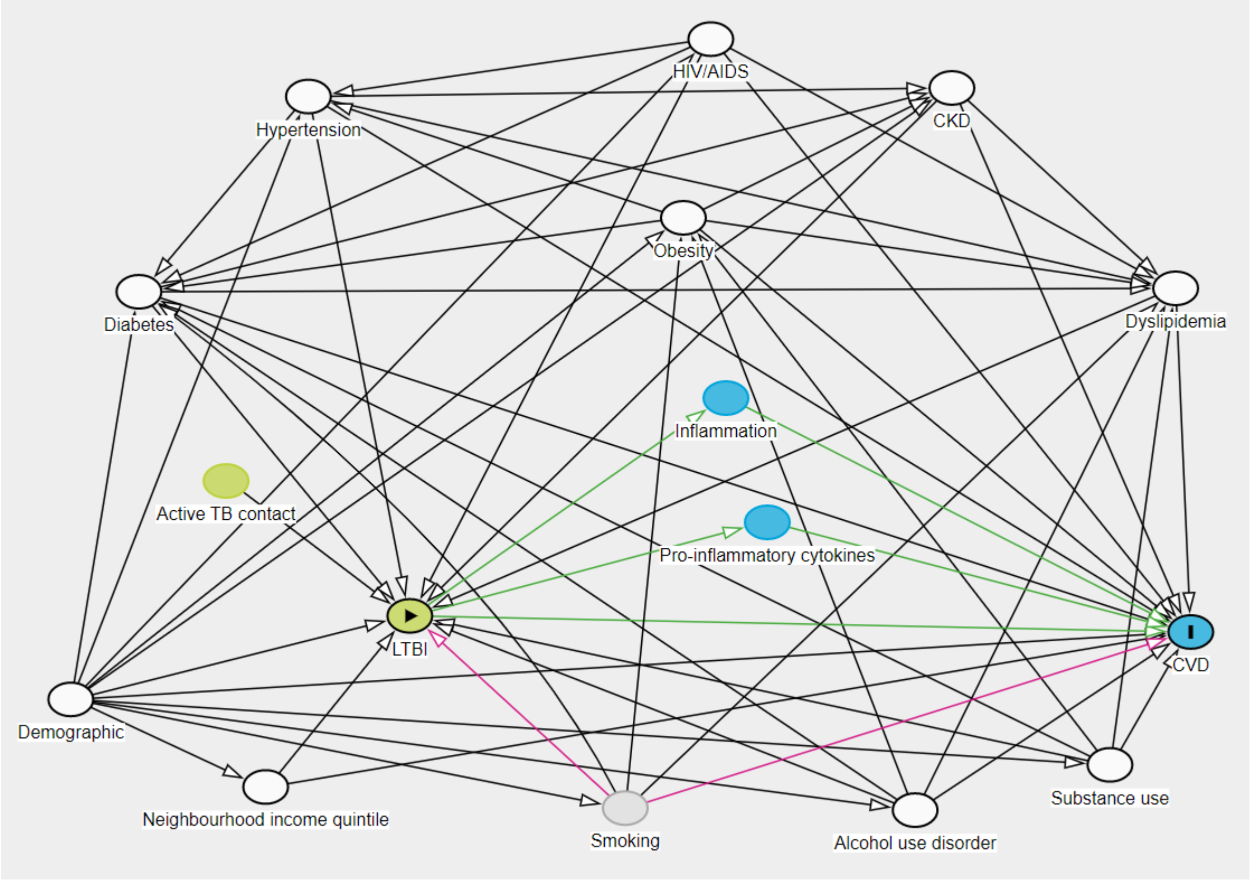

Appendix Figure 1: Causal diagram showing the relationship between LTBI (exposure variable) and time from cohort entry date to development of CVD (outcome variable) among people who immigrated to British Columbia, Canada, between 1985 and 2019. Here, demographic variables include age, sex, education, birth region, and immigration class. Smoking is an unmeasured confounder that creates biasing paths (red paths); active TB contact is an instrumental variable; inflammation and pro-inflammatory cytokines are mediators. Abbreviations – CKD: chronic kidney disease; CVD: cardiovascular disease; HIV/AIDS: human immunodeficiency virus/ acquired immunodeficiency syndrome; LTBI: latent tuberculosis infection; TB: tuberculosis.

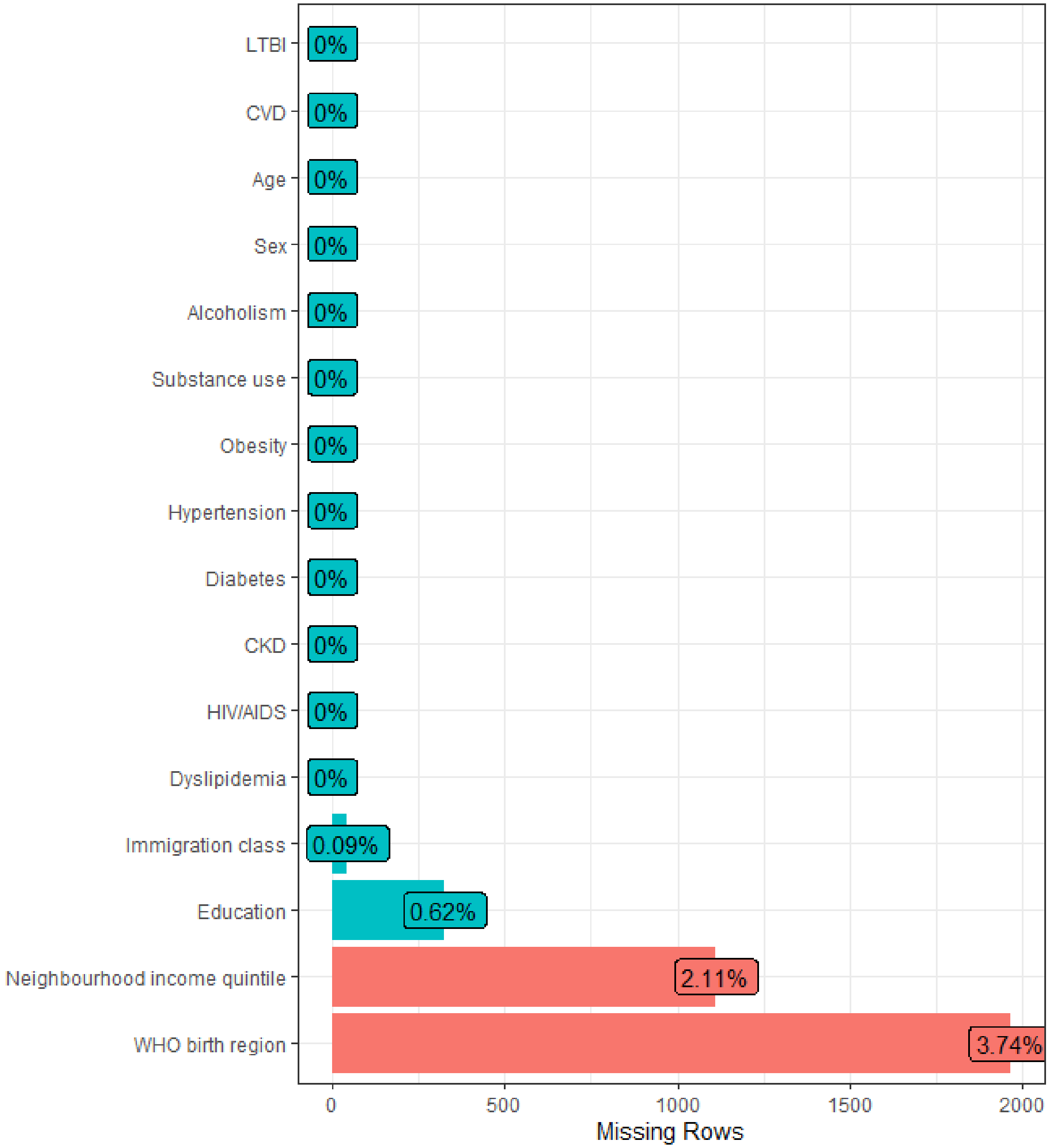

Appendix Figure 2: Percentage of missing values in a cohort of people who immigrated to British Columbia, Canada, between 1985 and 2019 and tested for latent tuberculosis infection (LTBI). Abbreviations – CKD: chronic kidney disease; CVD: cardiovascular disease; HIV/AIDS: human immunodeficiency virus/acquired immunodeficiency syndrome; LTBI: latent tuberculosis infection.

	Before	After	
**LTBI therapy**	**#CVD/PT**	**#CVD/PT**	**RR (95% CI)**
Not completed	50/18,705	78/24,645	1.18 (0.83-1.69)
Completed	65/25,433	82/32,544	0.99 (0.71-1.36)

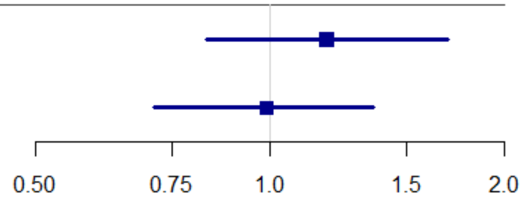

Appendix Figure 3: The rate ratio (RR) of cardiovascular disease (CVD) after the LTBI therapy completion (among those who completed therapy) or discontinuation (among those who did not complete therapy) compared to the rates from the same subjects before the latent tuberculosis infection (LTBI) test. The CVD rate ratio among those who completed the therapy was 0.99 (95% CI: 0.71-1.36) after the completion of LTBI therapy than before the LTBI test. On the other hand, the CVD rate ratio in people who did not complete LTBI therapy was 1.18 (95% CI: 0.83-1.69) after discontinuation of LTBI therapy than before the LTBI test. Here, 'before' means before the LTBI test; 'after' means after the LTBI therapy completion (among those who completed therapy) or discontinuation (among those who did not complete therapy); #CVD is the number of CVD events, PT is person-time in years, RR is the rate ratio; CVD is cardiovascular disease; LTBI is latent tuberculosis infection.

7. Appendix tables

Appendix Table 1: Characteristics of the people with and without information on latent tuberculosis infection (LTBI) therapy in a cohort of people who immigrated to British Columbia, Canada, between 1985 and 2019 and tested for LTBI (aim 2).

Characteristics	Have LTBI therapy information (N = 5,631)	No LTBI therapy information (N = 20,532)	SMD
Age at immigration in years, mean (SD)	20.69 (16.58)	19.39 (15.16)	0.082
Females, n (%)	3127 (55.5)	12440 (60.6)	0.103
Education, n (%)			0.103
None	348 (6.2)	1432 (7.0)	
Secondary or less	2426 (43.1)	7820 (38.1)	
Trade/diploma	1008 (17.9)	4073 (19.8)	
University degree	1849 (32.8)	7207 (35.1)	
Neighbourhood income quintile, n (%)			0.065
Lowest	2003 (35.6)	6785 (33.0)	
Low	1392 (24.7)	5027 (24.5)	
Middle	975 (17.3)	3678 (17.9)	
High	674 (12.0)	2646 (12.9)	
Highest	587 (10.4)	2396 (11.7)	
WHO birth region, n (%)			0.199
Africa	246 (4.4)	803 (3.9)	
Americans	289 (5.1)	1047 (5.1)	
Eastern Mediterranean	320 (5.7)	1226 (6.0)	
Europe	575 (10.2)	3188 (15.5)	
Southeast Asia	1325 (23.5)	3480 (16.9)	
Western Pacific	2876 (51.1)	10788 (52.5)	
Immigration class, n (%)			0.178
Economic	2752 (48.9)	11797 (57.5)	
Family	2238 (39.7)	6639 (32.3)	
Refugee	603 (10.7)	1922 (9.4)	
Other	38 (0.7)	174 (0.8)	
Alcohol use disorder, n (%)	158 (2.8)	596 (2.9)	0.006
Substance use, n (%)	154 (2.7)	585 (2.8)	0.007
Obesity, n (%)	392 (7.0)	1279 (6.2)	0.030
Hypertension, n (%)	175 (3.1)	361 (1.8)	0.088
Diabetes, n (%)	251 (4.5)	552 (2.7)	0.095
CKD, n (%)	19 (0.3)	18 (0.1)	0.054

HIV/AIDS, n (%)	75 (1.3)	199 (1.0)	0.034
Dyslipidemia, n (%)	45 (0.8)	137 (0.7)	0.015

Abbreviations – CKD: chronic kidney disease; CVD: cardiovascular disease; HIV/AIDS: human immunodeficiency virus/acquired immunodeficiency syndrome; LTBI: latent tuberculosis infection; SD: standard deviation; SMD: standardized mean difference; WHO: World Health Organization.

Appendix Table 2: Characteristics of the people who immigrated to British Columbia, Canada, between 1985 and 2019 and tested for latent tuberculosis infection (LTBI) using tuberculin skin test or interferon-gamma release assay, stratified by the LTBI therapy exposure status (aim 2).

Characteristics	LTBI negative (N = 23,034)	LTBI therapy		SMD
		Complete (N = 3,202)	Incomplete (N = 2,429)	
Age at immigration in years, mean (SD)	18.80 (16.06)	21.02 (16.72)	20.25 (16.38)	0.090
Females, n (%)	13629 (59.2)	1743 (54.4)	1384 (57.0)	0.064
Education, n (%)				0.156
None	2738 (11.9)	206 (6.4)	142 (5.8)	
Secondary or less	9925 (43.1)	1349 (42.1)	1077 (44.3)	
Trade/diploma	3732 (16.2)	578 (18.1)	430 (17.7)	
University degree	6639 (28.8)	1069 (33.4)	780 (32.1)	
Neighbourhood income quintile, n (%)				0.107
Lowest	7149 (31.0)	1132 (35.4)	871 (35.9)	
Low	5648 (24.5)	767 (24.0)	625 (25.7)	
Middle	4171 (18.1)	589 (18.4)	386 (15.9)	
High	3122 (13.6)	391 (12.2)	283 (11.7)	
Highest	2944 (12.8)	323 (10.1)	264 (10.9)	
WHO birth region, n (%)				0.232
Africa	848 (3.7)	140 (4.4)	106 (4.4)	
Americans	2301 (10.0)	158 (4.9)	131 (5.4)	
Eastern Mediterranean	1437 (6.2)	172 (5.4)	148 (6.1)	
Europe	3987 (17.3)	307 (9.6)	268 (11.0)	
Southeast Asia	4955 (21.5)	793 (24.8)	532 (21.9)	
Western Pacific	9506 (41.3)	1632 (51.0)	1244 (51.2)	
Immigration class, n (%)				0.083
Economic	12140 (52.7)	1561 (48.8)	1191 (49.0)	
Family	8901 (38.6)	1285 (40.1)	953 (39.2)	
Refugee	1805 (7.8)	335 (10.5)	268 (11.0)	

Other	188 (0.8)	21 (0.7)	17 (0.7)	0.049
Alcohol use disorder, n (%)	862 (3.7)	79 (2.5)	79 (3.3)	0.053
Substance use, n (%)	899 (3.9)	80 (2.5)	74 (3.0)	0.038
Obesity, n (%)	1469 (6.4)	203 (6.3)	189 (7.8)	0.024
Hypertension, n (%)	644 (2.8)	92 (2.9)	83 (3.4)	0.013
Diabetes, n (%)	953 (4.1)	145 (4.5)	106 (4.4)	0.014
CKD, n (%)	95 (0.4)	12 (0.4)	7 (0.3)	0.020
HIV/AIDS, n (%)	386 (1.7)	42 (1.3)	33 (1.4)	0.015
Dyslipidemia, n (%)	144 (0.6)	26 (0.8)	19 (0.8)	0.059

Abbreviations – CKD: chronic kidney disease; CVD: cardiovascular disease; HIV/AIDS: human immunodeficiency virus/acquired immunodeficiency syndrome; LTBI: latent tuberculosis infection; SD: standard deviation; SMD: standardized mean difference; WHO: World Health Organization.

Appendix Table 3: Complementary analyses for Aim 2 of exploring the relationship between completion of latent tuberculosis infection (LTBI) therapy and time from the cohort entry to first occurrence of cardiovascular disease (CVD) among people who immigrated to British Columbia, Canada, between 1985 and 2019.

Complementary analyses	HR (95% CI)	
	Complete LTBI therapy vs no LTBI	Incomplete LTBI therapy vs no LTBI
Dealing with changing the exposure status [1]		
Time-varying exposure exposure definition	1.03 (0.86-1.23)	1.24 (1.01-1.52)
Dealing with violations of the proportional hazards assumption [2]		
Modified Poisson regression	1.04 (0.88-1.24)	1.25 (1.03-1.50)

Abbreviations – CI: confidence interval; CVD: cardiovascular disease; HR: hazard ratio; LTBI: latent tuberculosis infection.

[1] The time-dependent Cox model was fitted with time-varying LTBI exposure status, adjusting for age at immigration, sex, neighbourhood income quintile, education, region of birth, immigration class, alcohol use disorder, substance use, hypertension, diabetes, chronic kidney disease, obesity, HIV/AIDS, and dyslipidemia.

[2] The modified Poisson regression with binary CVD outcome variable and an offset by the natural logarithm of follow-up time was fitted, adjusting for age at immigration, sex, neighbourhood income quintile, education, region of birth, immigration class, alcohol use disorder, substance use, hypertension, diabetes, chronic kidney disease, obesity, HIV/AIDS, and dyslipidemia. The 95% confidence interval was calculated using robust sandwich method.

8. Reporting checklist

	Item No.	Reporting of Studies Conducted Using Observational Routinely-Collected Health Data (RECORD) items	Location in manuscript where items are reported
Title and abstract			
	1	RECORD 1.1: The type of data used should be specified in the title or abstract. When possible, the name of the databases used should be included.	pp 1-2
		RECORD 1.2: If applicable, the geographic region and timeframe within which the study took place should be reported in the title or abstract.	pp 1-2
		RECORD 1.3: If linkage between databases was conducted for the study, this should be clearly stated in the title or abstract.	pp 2
Introduction			
Background rationale	2		
Objectives	3		
Methods			
Study Design	4		
Setting	5		
Participants	6	RECORD 6.1: The methods of study population selection (such as codes or algorithms used to identify subjects) should be listed in detail. If this is not possible, an explanation should be provided.	pp 3
		RECORD 6.2: Any validation studies of the codes or algorithms used to select the population should be referenced. If validation was conducted for this study and not published elsewhere, detailed methods and results should be provided.	pp 4
		RECORD 6.3: If the study involved linkage of databases, consider use of a flow diagram or other graphical display to demonstrate the data linkage	Fig. 1

		process, including the number of individuals with linked data at each stage.	
Variables	7	RECORD 7.1: A complete list of codes and algorithms used to classify exposures, outcomes, confounders, and effect modifiers should be provided. If these cannot be reported, an explanation should be provided.	Appendix pp 2-3
Data sources/ measurement	8		
Bias	9		
Study size	10		
Quantitative variables	11		
Statistical methods	12		
Data access and cleaning methods		RECORD 12.1: Authors should describe the extent to which the investigators had access to the database population used to create the study population.	pp 9
		RECORD 12.2: Authors should provide information on the data cleaning methods used in the study.	pp 3-4
Linkage		RECORD 12.3: State whether the study included person-level, institutional-level, or other data linkage across two or more databases. The methods of linkage and methods of linkage quality evaluation should be provided.	pp 3-4
Results			
Participants	13	RECORD 13.1: Describe in detail the selection of the persons included in the study (i.e., study population selection) including filtering based on data quality, data availability and linkage. The selection of included persons can be described in the text and/or by means of the study flow diagram.	pp 6, Fig. 1
Descriptive data	14		
Outcome data	15		

Main results	16		
Other analyses	17		
Discussion			
Key results	18		
Limitations	19	RECORD 19.1: Discuss the implications of using data that were not created or collected to answer the specific research question(s). Include discussion of misclassification bias, unmeasured confounding, missing data, and changing eligibility over time, as they pertain to the study being reported.	pp 8-9
Interpretation	20		
Generalisability	21		
Other Information			
Funding	22		
Accessibility of protocol, raw data, and programming code		RECORD 22.1: Authors should provide information on how to access any supplemental information such as the study protocol, raw data, or programming code.	pp 9

Note: Checklist is protected under Creative Commons Attribution (CC BY) license. Reference: Benchimol EI, Smeeth L, Guttmann A, Harron K, Moher D, Petersen I, Sørensen HT, von Elm E, Langan SM, RECORD Working Committee. The REporting of studies Conducted using Observational Routinely-collected health Data (RECORD) statement. PLoS Medicine. 2015;12(10):e1001885.

9. Appendix References

1. Tonelli M, Wiebe N, Fortin M, Guthrie B, Hemmelgarn BR, James MT, Klarenbach SW, Lewanczuk R, Manns BJ, Ronksley P. Methods for identifying 30 chronic conditions: application to administrative data. BMC Med Inform Decis Mak. 2016;15(31):1–11.
2. Quan H, Sundararajan V, Halfon P, Fong A, Burnand B, Luthi J-C, Saunders LD, Beck CA, Feasby TE, Ghali WA. Coding algorithms for defining comorbidities in ICD-9-CM and ICD-10 administrative data. Med Care. 2005;43(11):1130–9.
3. World Health Organization. Tuberculosis data [Internet]. 2022 [cited 2022 Jan 12]. Available from: https://www.who.int/teams/global-tuberculosis-programme/data
4. Ronald LA, Campbell JR, Balshaw RF, Romanowski K, Roth DZ, Marra F, Cook VJ, Johnston JC. Demographic predictors of active tuberculosis in people migrating to British Columbia, Canada: a retrospective cohort study. Can Med Assoc J. 2018;190(8):E209–16.
5. White IR, Royston P. Imputing missing covariate values for the Cox model. Stat Med. 2009;28(15):1982–98.
6. Zhang Z. Multiple imputation with multivariate imputation by chained equation (MICE) package. Ann Transl Med. 2016;4(2):30.
7. Kumamaru H, Schneeweiss S, Glynn RJ, Setoguchi S, Gagne JJ. Dimension reduction and shrinkage methods for high dimensional disease risk scores in historical data. Emerg Themes Epidemiol. 2016;13(1):1–10.
8. Karim ME, Pang M, Platt RW. Can we train machine learning methods to outperform the high-dimensional propensity score algorithm? Epidemiology. 2018;29(2):191–8.
9. Zhou Z, Rahme E, Abrahamowicz M, Pilote L. Survival bias associated with time-to-treatment initiation in drug effectiveness evaluation: a comparison of methods. Am J Epidemiol. 2005;162(10):1016–23.
